# HortDB V1.0: a genomic database of horticultural plants

**DOI:** 10.1093/hr/uhae224

**Published:** 2024-08-12

**Authors:** Zhidong Li, Chong Wang, Shenghao Wang, Wenquan Wang, Fei Chen

**Affiliations:** National Key Laboratory for Tropical Crop Breeding, College of Breeding and Multiplication (Sanya Institute of Breeding and Multiplication), Hainan University, Sanya 572025, China; College of Tropical Agriculture and Forestry, Hainan University, Danzhou 571737, China; National Key Laboratory for Tropical Crop Breeding, College of Breeding and Multiplication (Sanya Institute of Breeding and Multiplication), Hainan University, Sanya 572025, China; College of Tropical Agriculture and Forestry, Hainan University, Danzhou 571737, China; National Key Laboratory for Tropical Crop Breeding, College of Breeding and Multiplication (Sanya Institute of Breeding and Multiplication), Hainan University, Sanya 572025, China; College of Tropical Agriculture and Forestry, Hainan University, Danzhou 571737, China; National Key Laboratory for Tropical Crop Breeding, College of Breeding and Multiplication (Sanya Institute of Breeding and Multiplication), Hainan University, Sanya 572025, China; College of Tropical Agriculture and Forestry, Hainan University, Danzhou 571737, China; National Key Laboratory for Tropical Crop Breeding, College of Breeding and Multiplication (Sanya Institute of Breeding and Multiplication), Hainan University, Sanya 572025, China; College of Tropical Agriculture and Forestry, Hainan University, Danzhou 571737, China

Dear Editor,

Horticulture is a classical discipline that focuses on a wide array of plants, including fruits, vegetables, ornamentals, beverages, spices, and medicinal herbs. Unlike field crops, horticultural plants require controlled conditions and specific equipment for their growth [1] and consequently they share several biological characteristics, such as asexual reproduction and grafting [2], a long juvenile phase [3], various postharvest treatments of products [4], and unique secondary metabolism [5]. Over the years, significant amounts of omics data [6] and tools have accumulated for various horticultural plants [7, 8]. However, despite the construction of genomic databases for some individual plants [9] and the global recognition of horticulture as a classical discipline, a comprehensive horticultural plant-centric database is still lacking.

To fulfill the needs of comparative analyses in dry laboratories and experimental studies in wet laboratories, we have developed a user-friendly omics database for horticultural plants, utilizing Java 11, Python 3.8, HTML5, R 3.6, and JavaServer Pages scripts. This database, named HortDB V1.0 (https://bioinformatics.hainanu.edu.cn/hortdb/), serves as a dependable repository of genomic data for horticultural plants, alongside providing bioinformatic tools for researchers and breeders. HortDB is hosted on our dedicated server and operates with Apache Tomcat 8.5.47 as its web server. All pre-processed data are stored within the MySQL 5.7.30 database, accessible through the HortDB front-end web application. Genomic and transcriptomic data have been meticulously cataloged within HortDB. Importantly, all data and tools are freely accessible without requiring registration.

**Figure 1:**
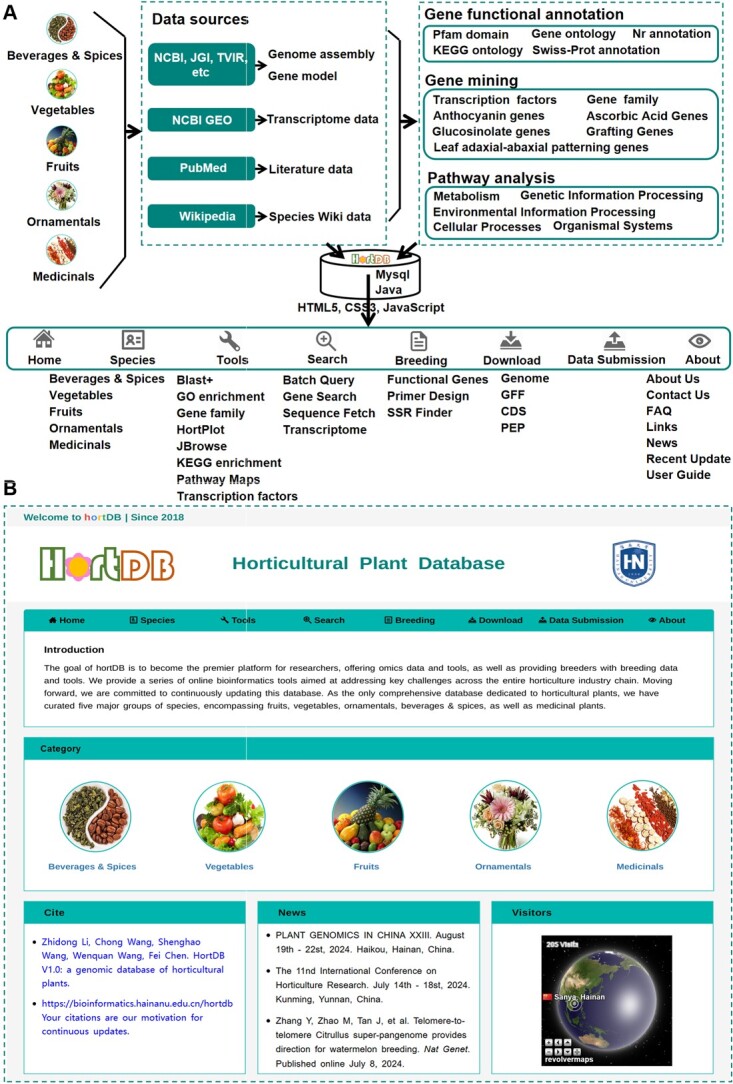
Workflow chart and homepage of HortDB. **A** Workflow of this database. **B** Homepage of HortDB.

HortDB V1.0 is committed to providing users with a comprehensive array of genomic data and tools for 174 horticultural plants (Table 1), and stores more genomic data than any other horticultural database [10]. The homepage of HortDB features a menu containing a range of tools, news updates, and helpful information (Fig. 1). Quick links to five categories of horticultural plants are conveniently provided on the homepage, facilitating easy navigation. Database news is prominently displayed on the HortDB homepage. To monitor user traffic and optimize website performance, we employ RevolverMaps to track visits to the database.

**Table 1:** Datasets included in HortDB.

**Item**	**Quantity**
Families/genera/species	99/126/174
Beverages and spices	8
Vegetables	52
Fruits	62
Ornamentals	20
Medicinals	32
Number of genomes	203
Genome size	72.78 Gb
Number of GFF files	201
Size of GFF files	2.07 Gb
Number of CDS files	203
Size of CDS files	3.46 Gb
Number of protein files	203
Size of protein files	2.27 Gb
Genes/proteins	9 450 975/10 015 360
PFAM families/TF families	19 629/63
GO/KEGG/NR/PFAM/Swiss-Prot	3 497 703/3 373 751/7 581 636/6 161 837/4 692 385
Horticulture related genes	17 777
Pathways	108
Species with JBrowse	170
Species with Blast+	174
Expression data sets	134
References	373

Currently, HortDB encompasses 174 horticultural plant species, comprising cultivated crops as well as their wild relatives. Each species features an introduction section containing background information, taxonomy, common names, and associated publications. For certain species, multiple genomic assemblies are available within HortDB; for instance, there are four genomic assemblies for apple and five for chrysanthemum. We aim for these extensive datasets, information resources, and tools to facilitate rapid understanding of each species and its associated data among researchers.

HortDB offers a comprehensive suite of built-in tools tailored for comparative genomic research purposes (Fig. 1). The Blast+ and Batch Query toolkit allows rapid comparison, visualization, and batch download of selected sequences. Additionally, tools such as gene search, transcription factors, and gene family search facilitate detailed exploration and annotation of genes. The JBrowse tool enables visualization and search functionalities for genes and sequences within assembled genomes. To streamline Gene Ontology (GO) and Kyoto Encyclopedia of Genes and Genomes (KEGG) enrichment analyses, HortDB integrates tools specifically designed for GO and KEGG enrichment. This feature encompasses 108 pathways associated with ‘Metabolism’, ‘Genetic Information Processing’, ‘Environmental Information Processing’, ‘Cellular Processes’, and ‘Organismal Systems’. The integrated HortPlot toolkit provides nine commonly used plotting functions for online visualization, including Venn diagrams, heat maps, Manhattan plots, violin plots, beeswarm plots, box plots, volcano plots, ternary plots, and bubble plots. Furthermore, the Sequence Fetch tool empowers users to extract sequences from specified genomic positions. Extensive mining efforts have been conducted across 41 horticulturally significant plants, identifying 17 777 related genes associated with categories such as ‘Anthocyanin Genes’, ‘Ascorbic Acid Genes’, ‘Glucosinolate Genes’, ‘Grafting Genes’, and ‘Leaf Adaxial-abaxial Patterning Genes’. In addition to these functionalities, HortDB offers online primer design and SSR mining capabilities. Our goal is to provide analytical tools within HortDB that facilitate researchers in effectively harnessing genomic data related to horticultural crops.

HortDB aspires to stand as a premier platform for horticulture research. All data within HortDB are openly accessible to the public, encompassing news updates, conference details, researcher profiles, and external links to related databases. Additionally, 373 genome publications have been meticulously collected and made available. The homepage of HortDB hosts an annual bluebook detailing advancements in genome sequencing of horticultural plants. To aid users, frequently asked questions (FAQs) and a comprehensive user guide are provided for reference and assistance.

In summary, HortDB serves as a comprehensive platform housing a wealth of available omics data for horticultural plants, complemented by a suite of built-in bioinformatics tools. Moving forward, HortDB is committed to continuously updating its data and tools to address the growing demand for comparative genomics in the era of big data. With the aim of becoming a central hub for data collection and online analyses in horticulture research, we envision HortDB expanding its repository and toolkit significantly in the coming years. HortDB actively encourages data sharing among users and warmly welcomes valuable suggestions for enhancements from researchers worldwide. By collaborating to collectively maintain and improve HortDB, we aim to foster a vibrant and dynamic research community dedicated to advancing horticulture research.
